# Implementation of sodium alginate-Fe_3_O_4_ to localize undiagnosed small pulmonary nodules for surgical management in a preclinical rabbit model

**DOI:** 10.1038/s41598-022-13884-w

**Published:** 2022-06-15

**Authors:** Zhi-xuan Zhang, Lu Lv, Ai-hua Shi, Yun-hao Li, Tian-ren Wang, Yuan-hang Guo, Bao-juan Hu, Xiao-peng Yan, Jun-ke Fu, Feng Ma, Hao-hua Wang, Yi Lv, Yong Zhang

**Affiliations:** 1 Department of Thoracic Surgery, The First Affiliated Hospital of Xi’an Jiaotong University, Xi’an, 710061 China; 2 National Local Joint Engineering Research Center for Precision Surgery and Regenerative Medicine, The First Affiliated Hospital of Xi’an Jiaotong University, Xi’an, 710061 China; 3 Xi’an Jiaotong University Health Science Center, Xi’an, 710061 China; 4 Xi’an Chest Hospital, Xi’an, 710061 China; 5 Department of Hepatobiliary Surgery, The First Affiliated Hospital of Xi’an Jiaotong University, Xi’an, 710061 China; 6 Department of Thoracic Surgery, National Local Joint Engineering Research Center for Precision Surgery and Regenerative Medicine, The First Affiliated Hospital of Xi’an Jiaotong University, 277 Yanta West Road, Xi’an, 710061 Shaanxi China

**Keywords:** Respiratory tract diseases, Implants, Lung cancer

## Abstract

Many methods are used to locate preoperative small pulmonary nodules. However, deficiencies of complications and success rates exist. We introduce a novel magnetic gel for small pulmonary nodules localization in rabbit model, and furtherly evaluate its safety and feasibility. Rabbits were used as the experimental objects. A magnetic gel was used as a tracer magnet, mixed as sodium alginate-Fe_3_O_4_ magnetic fluid and calcium gluconate solution. In short-term localization, a coaxial double-cavity puncture needle was applied to inject the gel into the lung after thoracotomy, and a pursuit magnet made of Nd-Fe-B permanent magnetic materials was used to attract the gel representing location of the nodule. In long-term localization, the gel was injected under X-ray guidance. Imaging changes to the lung were observed under X-ray daily. Thoracotomy was performed to excise tissue containing the gel, and hematoxylin–eosin staining was used to observe the tissue on postoperative days 1, 3, 5, and 7. Observe tissues morphology of heart, liver, spleen, and kidney in the same way. The gel was formed after injection and drew lung tissue to form a protrusion from the lung surface under the applied magnetic field. No complication was observed. The shape and position of the gel had not changed when viewed under X-ray. Pathological analysis showed the gel had a clear boundary without diffusion of magnetic fluid. All tissues retained good histologic morphology and no magnetic fluid was observed. Our study preliminarily suggested that the technique using sodium alginate-Fe_3_O_4_ magnetic gel to locate small pulmonary nodules with guidance of X-ray, and to search for them under an applied magnetic field during the operation is safe and feasible.

## Introduction

With the development of computed tomography (CT) technology, the clinical detection rate of small pulmonary nodules (SPNs) is increasing. SPNs are principally peripheral solitary or multiple small (5–10 mm in diameter) lesions present in the lungs, without atelectasis, hilar lymph node enlargement, or pleural effusion^1,2^. Their probability of malignancy is 3.3–10.8%^3,4^, and surgical treatment is accepted as the first choice in clinical practice. Due to the small volume and unclear boundary of small pulmonary nodules, it is difficult to be seen or detected by the naked eye during the operation, and the collapse of the lung during the operation will also increase the difficulty of finding small pulmonary nodules, and thus, the localization of SPNs before surgery is necessary^5,6^. The traditional SPNs localization technique prior to surgery is CT-guided percutaneous puncture localization, such as hook-wire localization and micro-coil localization^7^. However, the incidence of pneumothorax, hemothorax, and other complications using these methods is high^8–10^. Therefore, exploring a better localization method of SPNs with a higher success rate and fewer complications is important.

The magnetic tracer technique (MTT) is an innovative diagnostic and treatment method, which places a tracer magnet into the human body and uses the magnetic force between magnets to search for the position of the tracer magnet with a pursuit magnet outside the body^11^. Currently, MTT has been applied to trace the sentinel lymph nodes of breast cancer in the clinical^12^, and has been applied in a variety of animal models of digestive tract tumors such as esophagus, stomach, colorectal^13–15^. The pursuit magnet of MTT is usually a permanent magnet composed of Nd-Fe-B. The tracer magnet for MTT can be solid magnet or magnetic fluid. However, for the small pulmonary nodules 1 cm below the visceral pleura, the solid magnet is difficult to access, and the magnetic fluid is easy to diffuse, which is difficult to achieve the positioning effect. When takes sodium alginate solution as the base fluid of the magnetic fluid, it can be crosslinked with Ca^2+^ to form a gel microsphere with a diameter of millimeter size, which confines the magnetic particles to gel to accurately locate the SPNs.

We aimed to explore a method for localization of SPNs based on magnetic tracer technology and magnetic gel, which can make up for the shortcomings of existing methods, improve the success rate and reduce complications. In this experiment, we verify its feasibility and safety through a rabbit model.

## Discussion

Statistics from the American Cancer Society show that patients with local, regional, and distant metastasis at first diagnosis of lung cancer have corresponding 5-year relative survival rates of 56%, 29%, and 5%, respectively^16^. As SPNs tend to malignancy^3,17^, their early diagnosis is important. With the application of low-dose chest CT technology becoming increasingly common, the detection rate of SPNs has increased. As mentioned above, accurately locating SPNs during surgery is difficult and a known location before surgery is preferable^5^. At present, there are many methods for preoperative localization of SPNs, but these each has their own shortcomings, described as follows: (1) CT-guided percutaneous puncture localization, such as hook-wire localization and micro-coil localization has a blind spot for placement, metal markers are easily dislocated, and the technique has a high occurrence rate of complications such as pneumothorax and hemothorax^18–20^; (2) Navigation bronchoscopy technology requires large amount of equipment, and the operative time is long; (3) Ultrasound-assisted localization requires the lung tissue to reach a state of complete atelectasis and the extent of the lesion must be limited to 2 cm below the visceral pleura; (4) In the dye injection localization method, the dye is easily diffused, which expands the localization range and causes difficulties with recognition on the pigmented lung surface^21^. In addition, all these technologies require surgery immediately after positioning, which is a great challenge for the surgical arrangement and management of the hospital.

In order to improve the success rate of operation, reduce complications and relieve the pressure of operation arrangement, we designed a new method based on the MTT, which used sodium alginate-Fe_3_O_4_ magnetic fluid and calcium gluconate solution to form a magnetic gel in the lungs, and a pursuit magnetic field that was applied to the lung surface for targeted localization.

The MTT device included two parts: a tracer magnet and a pursuit magnet. We used Nd-Fe-B as the material for the pursuit magnet in this experiment. Nd-Fe-B is a high magnetic energy product that has been used commonly in magnetic surgery^22^. It has an excellent attraction and localization effect, which is convenient and economical in surgery. The main component of the tracer magnet, the magnetic fluid, is a new material produced by the intersection of physics, chemistry, nanotechnology, and other fields, and it is composed of permanent magnetic particles, liquid carrier, and dispersant^23^. The micron-sized Fe_3_O_4_ permanent magnetic particles used in this experiment have superparamagnetism. They move randomly in the liquid carrier without an applied magnetic field and have no magnetization intensity. When a magnetic field is applied, the magnetic moment of the permanent magnetic particles conforms to the orientation of the magnetic field line. Its magnetic force is revealed in response to change in the applied magnetic field^24^. Sodium alginate, a high-molecular-weight polysaccharide, can be used as both a liquid carrier and a dispersant. Fe_3_O_4_ permanent magnetic particles are uniformly distributed under the sodium alginate solution coating. They will not interact and agglomerate in the normal state, and they have both solid magnetic properties and liquid fluidity. In addition, the sodium alginate solution can be solidified to form a clumpy magnetic gel^25^ after contact with Ca^2+^ in the curing agent, which can achieve targeted localization under an applied magnetic field.

Compared with the previous methods, our method has a higher success rate, fewer complications, more simple operation, lower costs, and more flexible operation arrangement. The most commonly used hook-wire localization and dye localization are taken as examples.

Compared with hook-wire localization, our method has fewer complications and lower costs: the tip of hook-wire is in the lung and the tail is outside the chest wall. Dislocation of the hook is easy to happen when coughing and changing posture. At this time, the sharp hook will damage the lung tissue, cause pneumothorax and hemothorax, and even switch to thoracotomy. However, we made the magnetic fluid to form a gel completely inside the lung, which was closely combined with lung, without the risk of dislocation. In addition, we withdrew the needle while injecting. The formed gel can fill the needle path, with fewer complications such as pneumothorax and hemothorax. Compared with dye localization, such as methylene blue, our method has a higher positioning success rate and accuracy. Dye is easy to diffuse, which reduces the positioning accuracy, and for the pigmented lungs of smoking patients, dye cannot be used. Our method allows black magnetic particles to be confined to gel without dispersion. For pigmented lungs, they can also be found by magnetic attraction. Furthermore, the existing methods require surgery on the same day after positioning, which has high requirements for hospital operation arrangement. Our long-term experiments have verified that the magnetic gel can exist stably in the body for at least 7 days, enabling doctors to flexibly arrange the operation, avoiding the shortage of operating room.

Through our experimental results and existing studies, it can be explained that the method has biosafety for human body. Firstly, in terms of composition, it is composed of three parts: micron-sized Fe_3_O_4_ magnetic powder, sodium alginate solution and calcium gluconate solution: they all have high biosafety. Sodium alginate can stably exist in the human body, as it is similar to the extracellular matrix and can be degraded into non-toxic polysaccharides. It is widely used in drug delivery, wound repair, tissue engineering, and interventional therapy^26–29^. Ten percent calcium gluconate injection, as a prevalent calcium supplement in clinical practice, has a good safety record. In addition, Ca^2+^ can decrease the permeability of capillaries and increase their compactness^30^, which makes it more difficult for magnetic fluid to enter the circulation. Micron sized magnetic particles have been proved to be non-toxic to human cells such as lung epithelial cells, hepatocytes, islet cells and cartilage progenitor cells^31–34^, and have been safely used in various molecular cell imaging technologies^35,36^. McAteer et al^37^. Found that the magnetic powder was removed rapidly from the blood in the mice model. The magnetic powder was distributed in the lung, spleen, liver and kidney 30 min after entering the blood. After 24 h, the magnetic powder was removed from the kidney and lung and only existed in the liver and kidney. And These particles are internalized mainly involves the clathrin-dependent endocytic pathway^35^. Secondly, in our method, the two solutions (sodium alginate-Fe_3_O_4_ magnetic fluid and calcium gluconate solution) are injected into the lung to form a gel immediately, which will be removed with the lesion in the subsequent resection of SPNs. The gel was confined to the lesion and did not enter the blood circulation during the whole operation. According to the anatomical, imaging, and histological results of our short-term and long-term localization, black magnetic fluid was not observed in the other lung tissue except at the injection site. The radiographic results showed the position of the magnetic gel neither obviously changed under X-ray nor moved with respiration and blood flow; the boundary was clear. No magnetic fluid was observed in the remaining lung tissue. The hematoxylin–eosin staining results showed that the magnetic gel had a clear boundary without diffusion. No magnetic fluid was observed in hematoxylin–eosin stained tissues of heart, liver, spleen, or kidney. If magnetic gel remains in the lung tissue for 3 days or more, local tissue fibrosis can occur. This is because the magnetic gel causes local tissue destruction and inflammatory damage, although this does not affect the survival of animals, and the magnetic gel and surrounding fibrotic tissue will be resected during surgery. Irrespective of observation in the short-term (15 min) or long-term (7 days), the magnetic fluid in our study did not diffuse and existed stably in the lung tissue, preliminarily showing that it is safe to use sodium alginate-Fe_3_O_4_ magnetic gel in SPNs localization technology.

In the magnetic force test, when the distance between the magnetic gel and the pursuit magnet is greater than 10 mm, the magnetic force is almost zero. For the electronic products or metal products, the distance between the magnetic gel in the lung and them is far greater than 10 mm, and the magnetic gel will only exist in the human body for a few days, and then it will be removed with small pulmonary nodule resection. Therefore, our method will not have an impact on electronic devices such as mobile phones and people's lives.

Our in vitro experiments proved that the sodium alginate magnetic fluid immediately formed a gel after contact with the curing agent. The maximum magnetic force between the magnetic gel and the pursuit magnet was 0.22 N, which is similar to the maximum anchoring force of the traditional micro-coil method reported in the literature (0.20 N)^38^. The force was sufficient to make a tuberous part protrude from the lung surface without causing the magnetic gel to fall away. In the in vivo experiments, the localization function was effective. In addition to observing the SPNs protruding from the lung surface, the surgeon could feel the attraction between the magnetic gel and the pursuit magnet while holding the instrument. No pneumothorax or other complications occurred during the operation.

The magnetic particles were localized in the lung tissue in a gelatinous state, and the gel did not displace with movement of the lung. The animal experiment results showed that the magnetic fluid preparation process is simple and low in cost, the magnetic gel is easy to manipulate during the procedure to locate SPNs, and the intraoperative localization effect is significant. Compared with the placement of metal markers, there is no risk of detachment. Compared with dye injection, the technique is not limited by lung color deposition and other factors. The injection method of removing the needle while injecting is used to form a gel that can fill the needle path, which greatly reduces the complexity of the operation, reducing bleeding, pneumothorax, and other complications. Moreover, in the long-term localization, the magnetic gel was stable in the body for at least 7 days, suggesting the operation schedule can be flexible.

However, our experiment still had some limitations. For example, the use of a syringe with coaxial double-cavity puncture needle may be difficult for insufficiently trained doctors. The coaxial double-cavity puncture needle needs two hands to push the piston of calcium gluconate solution and magnetic fluid at the same time, and even an assistant to fix the syringe. The doctor who first came into contact with the syringe may not cooperate well with his assistant, or push the magnetic fluid too fast to enter the lung tissue before the calcium gluconate solution, resulting in the diffusion and unable to achieve the positioning effect. However, this injection method can be well mastered after several times of practice. Besides, there was fibrosis tissue around the location of magnetic gel in the lung, therefore we didn't observe the pathological changes over a longer period of time. These problems will be studied in our follow-up work.

## Materials and methods

### Ethics declarations

The experiment was approved by the Animal Experiment Ethics Committee of Xi’an Jiaotong University (Approval No. XJTULAC2020-1176). The animal experiments were conducted in compliance with the standard ethical guidelines under the control of the ethics committee mentioned above and the ARRIVE guidelines.

### Experimental animals

Fifteen adult New Zealand rabbits, weighing 2 to 4 kg and of either sex, were used for the experiments. The rabbits were housed in individual cages in a room with constant humidity and temperature. These healthy rabbits were allowed to accommodate to the environment for 1 week. The animal rooms were set at a 12-h light–dark cycle. The animals were provided by the Experimental Animal Center of the Medical Department of Xi’an Jiaotong University, Xi’an, China.

### Pursuit magnet

A cylindrical pursuit magnet (purchased from Shenzhen Lala Magnetic Materials Development Co., Ltd., Shenzhen, China) with a diameter of 10 mm and height of 3 mm was machined with sintered N45 neodymium iron boron (Nd-Fe-B) permanent magnet material. The magnetic flux density of the pursuit magnet was 13.2–13.8 kG and its maximum energy product was 342–366 kJ/m^3^. The magnets were radially saturated with magnetization, nickel-plated on the surface, sealed, and then sterilized with ethylene oxide.

### Tracer magnet

The tracer magnet comprised magnetic gel, formed by sodium alginate-Fe_3_O_4_ magnetic fluid and curing agent. The selected curing agent was medical 10% Calcium Gluconate Injection (China Drug Quantifier H51023153, Sichuan Meida Kanghuakang Pharmaceutical Co., Ltd., Chendu, China).

The magnetic fluid was composed of sodium alginate solution and micron-sized Fe_3_O_4_ magnetic powder (both purchased from Shanghai Aladdin Biochemical Technology Co., Ltd., Shanghai, China). A quantity of sodium alginate powder and deionized water were weighed and stirred until the sodium alginate was completely dissolved to form sodium alginate solutions of 1%, 2%, 2.5%, 3%, and 4% by mass concentration. The sodium alginate solutions were mixed with equal doses of curing agent to form a gel, observing the gel formation time, strength, condition, and texture. The gel strength was measured using an MCR302 rheometer (Anton Paar GmbH, Graz, Austria). A suitable concentration of sodium alginate was selected as the base solution, to which an amount of Fe_3_O_4_ magnetic powder was added and stirred well to obtain sodium alginate-Fe_3_O_4_ magnetic fluid suspension.

**Figure 1:**
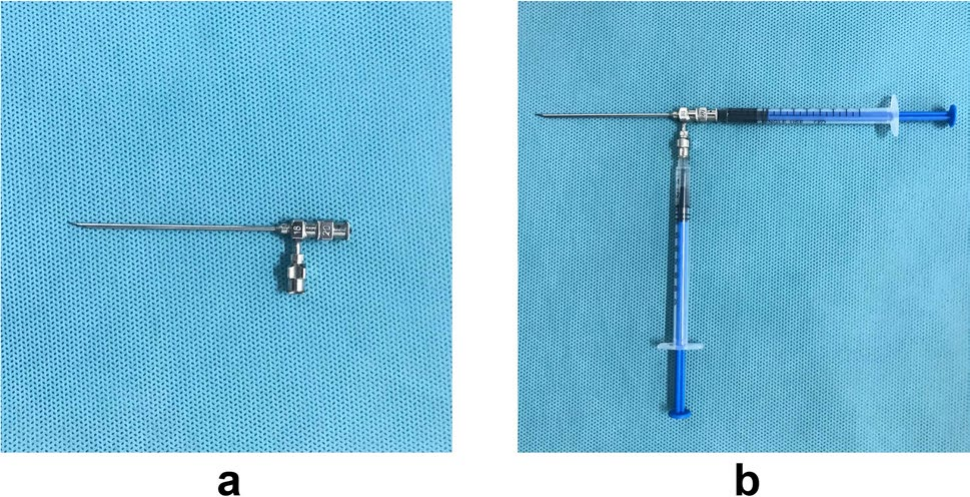
(**a**) Coaxial double-cavity puncture needle (inner cavity: 20 G; outer cavity: 16 G). (**b**) Operation method: The needle is divided into two parts, an inner and an outer cavity. The horizontal syringe contains the magnetic fluid, connected with the inner cavity; the longitudinal syringe contains the calcium gluconate solution, connected with the outer cavity. The two syringes are injected at the same time, and the magnetic fluid in the inner cavity enters the lung tissue under the calcium gluconate solution to form gel.

A 16–20 G coaxial double-cavity puncture needle (Fig. 1, Hefei Sipin Technology Co., Ltd., Hefei, China) was used to inject 0.1 mL magnetic fluid of different concentrations and 0.1 mL curing agent simultaneously. After the gel was formed, the gel properties were measured again using an MCR302 rheometer to compare the mechanical properties of the gel before and after the addition of magnetic powder, and the attraction between the pursuit magnet and the magnetic gel was measured using a UTM6202 electronic universal testing machine (Shenzhen Sansi Zongheng Technology Co., Ltd., Shenzhen, China).

### Short-term localization

Three rabbits were injected intravenously with 3% sodium pentobarbital 1 mL/kg at the ear margins, with the animals immobilized in the prone position. After skin preparation, they underwent a neck tracheotomy to establish an artificial airway, and then they were placed in the right lateral decubitus position. A thorax incision was made through the fifth intercostal space. The coaxial double-cavity puncture needle was used to inject 0.1 mL 10% calcium gluconate solution and 0.1 mL sodium alginate-Fe_3_O_4_ magnetic fluid simultaneously into the lower lobe of the left lung at the location of a hypothetical small pulmonary nodule. After the injection, the lung lobe was returned to its original position.

#### Observation of gross specimen

The lung tissue of the injection site was observed visually for hemorrhage, hematoma, and black magnetic gel morphology, and the degree of curing of the magnetic gel was ascertained by manual inspection using a finger. An applied magnetic field was provided by the pursuit magnet to observe whether the lung tissue could be attracted to protrude from the surface of the lung. The magnitude of attraction between the magnetic gel and the pursuit magnet was adjusted. The lung was observed for tissue tearing and damage, and whether the tracer magnet had protruded. The attracted lung tissue was where the hypothetical lung nodules were located.

#### Displacement of the magnetic gel

The lung tissue was placed back into the chest cavity and moved to different positions; mechanical ventilation was continued and changes in the magnetic gel’s position were observed in the ventilated state after passive displacement of lung tissue.

#### Diffusion of the magnetic fluid

The morphology and boundary of the magnetic gel were observed using X-ray 15 min after the injection. Lung tissue at the injection site and heart, liver, spleen, and kidney tissues were collected and fixed with 10% neutral formalin solution for 24 h at 4 °C and embedded in paraffin. The tissues were then stained with hematoxylin–eosin in 5 μm thickness slices. The shape and boundary of the magnetic gel and cell morphology of the surrounding tissue were examined under light microscope. The remaining lung tissue was dissected to the lung hilum to observe whether the injected magnetic fluid had diffused and the extent of diffusion.

### Long-term localization

Twelve rabbits were randomly divided into four groups (n = 3). For each rabbit, after local anesthetic puncture, a hypothetical SPN site was reached with the coaxial double-cavity puncture needle through the fifth intercostal space in the posterior axillary line under X-ray guidance while the plunger of the syringe was withdrawn. If there was no blood return, 0.1 mL magnetic fluid and 0.1 mL 10% calcium gluconate solution were injected at the same time. The magnetic gel was observed under X-ray for changes in size, boundary, and position. After the operation, the animals were observed for coughing and shortness of breath, and the morphology of the magnetic gel was observed under X-ray every 24 h.

The four groups of rabbits were separately operated on days 1, 3, 5, and 7 after the previous procedure as follows. They were injected intravenously with 1 mL/kg 3% pentobarbital sodium at the ear margin, immobilized in the right lateral position and skin preparation was performed. The chest was incised through the fifth intercostal space. A pursuit magnet was used to search for the magnetic gel on the surface of the lung. The attracted lung tissue acted as the imaginary SPN. The rabbits were euthanized and the tissue at the injection site and tissues of the heart, liver, spleen, and kidney were stained with hematoxylin–eosin. The shape, boundary, and surrounding tissue cell morphology of the magnetic gel were examined using a light microscope.

## Results

### Determining magnetic gel concentration

**Figure 2:**
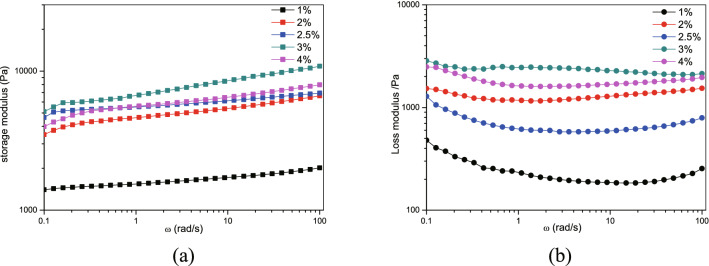
Properties of the magnetic gel at different concentrations. (**a**) Storage modulus. (**b**) Loss modulus.

The sodium alginate magnetic fluid and calcium gluconate solution formed a gel immediately when injected simultaneously. However, the gel formed with 1% sodium alginate was weak in strength, flocculent, and deformed easily when touched. It had no fixed shape when observed visually and touched by finger. The gel formed with 2%, 2.5%, 3%, and 4% sodium alginate had more strength and a more fixed shape, and did not deform with external force. Gel properties, measured using an MCR302 rheometer, are shown in Fig. 2. Compared with others, the gels formed by 2.5% sodium alginate were found to have higher storage modulus and lower loss modulus, indicating that its crosslinking density was larger and mechanical properties were better than the other concentrates. Therefore, a 2.5% concentration of sodium alginate was selected as the base solution.

**Figure 3:**
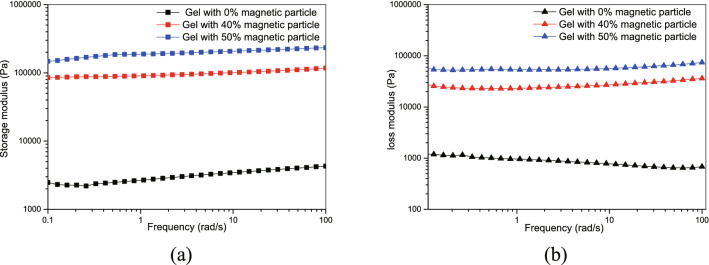
Comparison of gel properties before and after adding magnetic powder. (**a**) Storage modulus. (**b**) Loss modulus.

We added Fe_3_O_4_ magnetic powder to 2.5% sodium alginate solution. When the mass concentration of the magnetic powder was higher than 50%, the solution was not enough to infiltrate the surface of the magnetic powder to form a uniform and stable suspension. The gel properties were measured using an MCR302 rheometer. The results (Fig. 3) showed that the mechanical properties of the gel were not damaged after the addition of magnetic powder.

**Figure 4:**
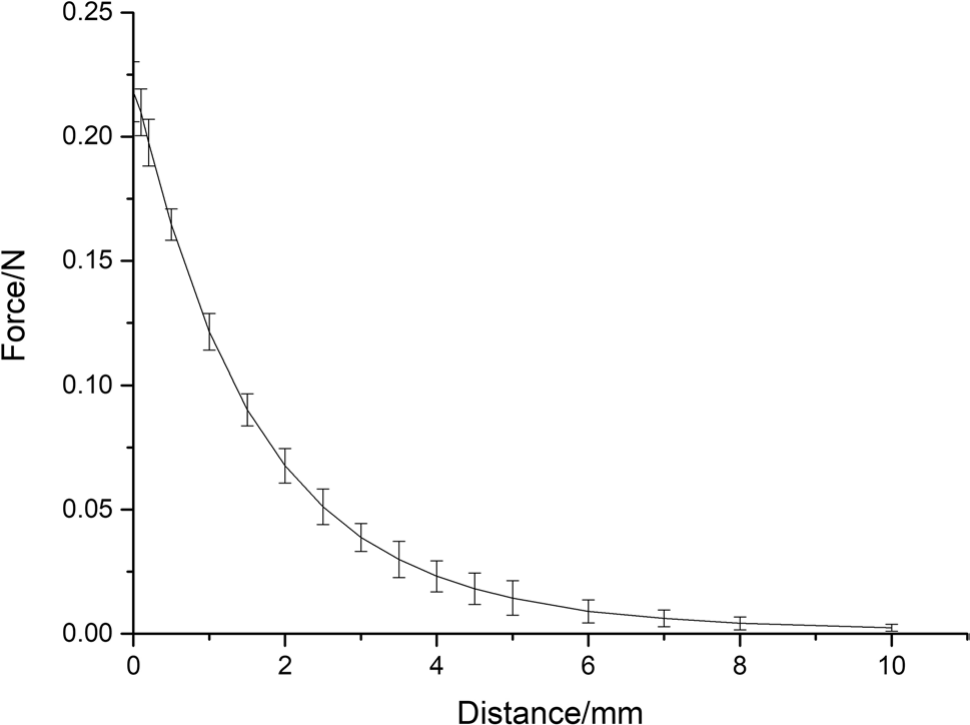
Change in magnetic force with distance between 0.2 mL magnetic gel (50% magnetic particle content, 3% sodium alginate) and the pursuit magnet (10 mm in diameter, 3 mm in height).

The magnetic force between 0.2 mL magnetic gel and the pursuit magnet (10 mm in diameter, 3 mm in height) was measured using a UTM6202 electronic universal testing machine. The pursuit magnet could provide a maximum magnetic force of 0.22 N. The difference in magnetic force between the magnetic gel and the pursuit magnet caused by the changes in distance is shown in Fig. 4.

In summary, to provide the most suitable magnetic force for the experiment, we used magnetic fluid with 2.5% sodium alginate and 50% magnetic particle content as the base liquid, 10% calcium gluconate solution as the curing agent, and a pursuit magnet 10 mm in diameter and 3 mm in height.

### Results of short-term localization

#### X-ray observation of magnetic gel

**Figure 5:**
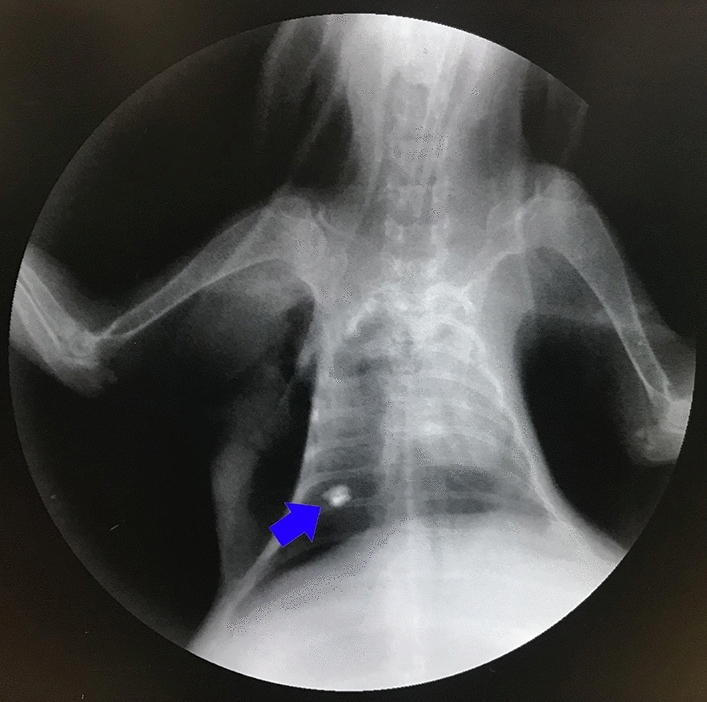
Magnetic gel in lung tissue (blue arrow) observed under X-ray.

All three rabbits in the group assigned to the short-term localization were successfully injected with magnetic gel to locate the imaginary lung nodules. No diffusion of magnetic fluid or displacement of the magnetic gel was observed under X-ray (Fig. 5).

#### Observation of gross specimens

**Figure 6:**
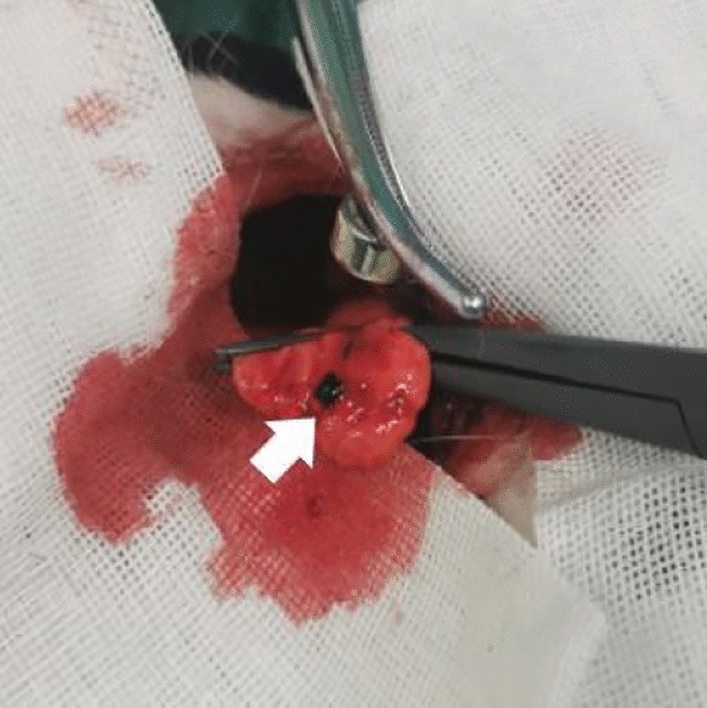
Magnetic gel in lung tissue of short-term localization (white arrow).

When observing lung tissue visually, the injection site was seen to contain black magnetic fluid without diffusion after injection. A small amount of bleeding with no hematoma was observed. The magnetic fluid solidified in the lung to form a mass of gel. Its shape and position did not change with external force when the injection site was touched with the tip of the forefinger (Fig. 6).

**Figure 7:**
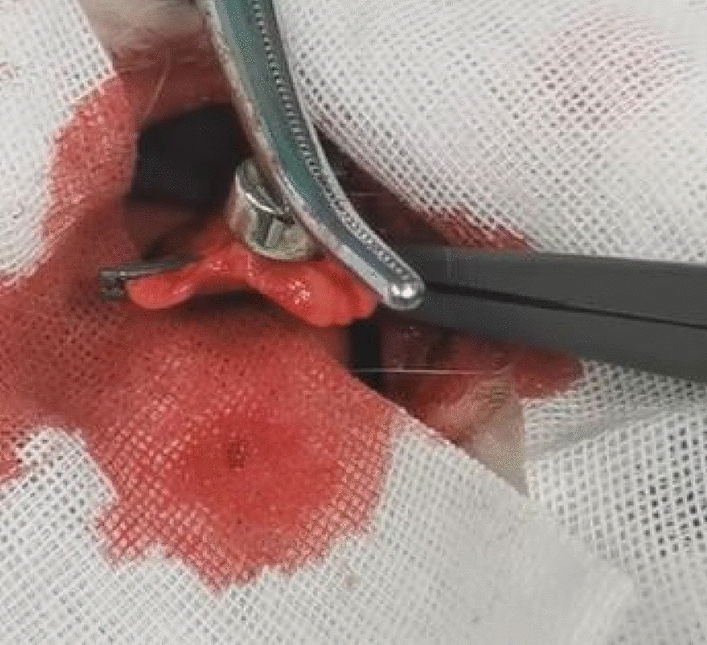
Localization effect under applied magnetic field.

A pursuit magnet was used to provide an applied magnetic field that could attract the magnetic gel, and this drew the lung tissue together to protrude from the lung surface (Fig. 7). No tearing or wound was observed in the lung tissue and no magnetic gel escaped after removal of the pursuit magnet.

#### Movement of the magnetic gel

The magnetic gel remained at the initial injection site without significant displacement after spontaneous breathing, mechanical ventilation, and passive displacement of lung tissue.

#### Diffusion of magnetic fluid

**Figure 8:**
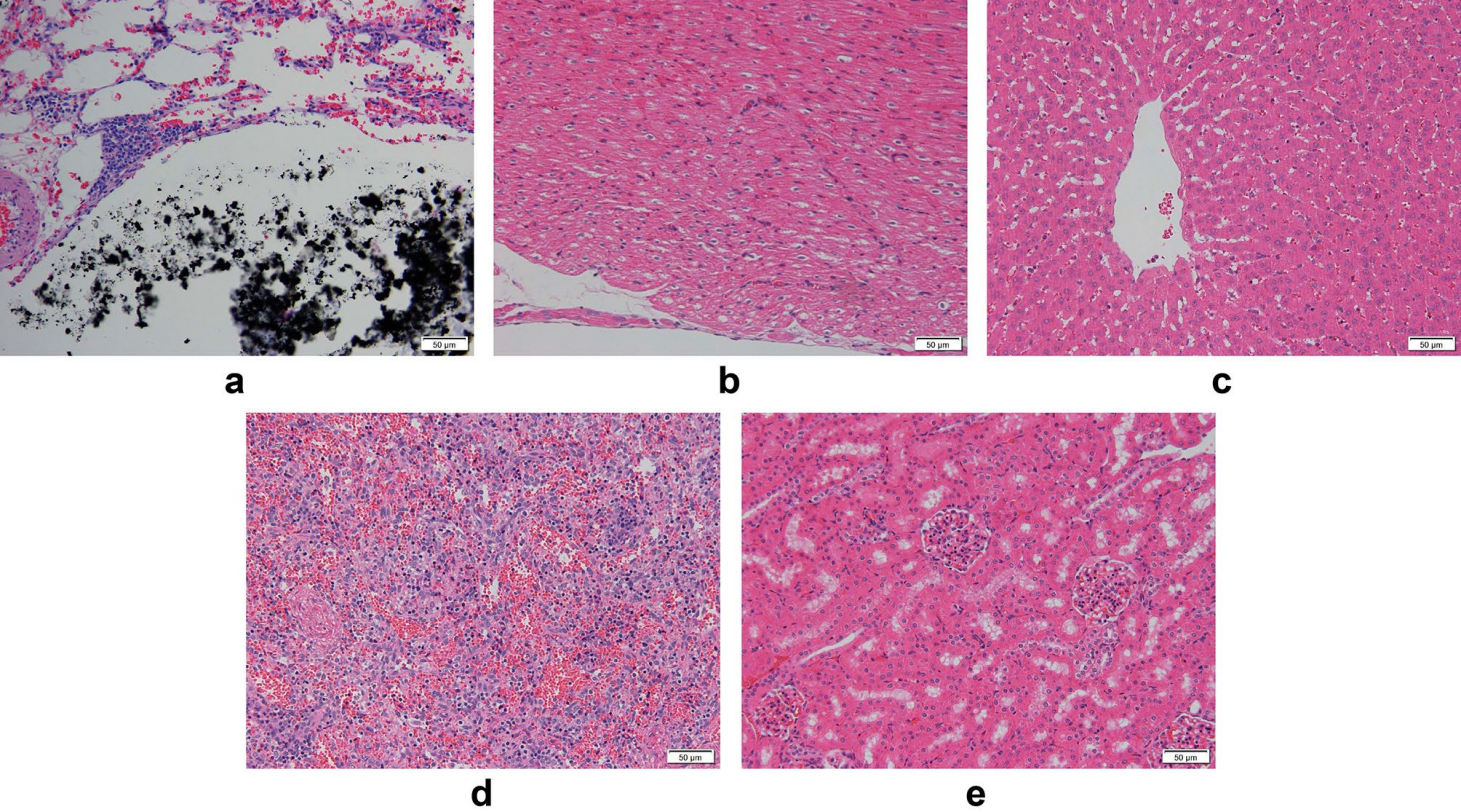
(**a**) Magnetic gel in lung tissue 15 min after injection, (**b**–**e**) Morphology of (**b**) heart, (**c**) liver, (**d**) spleen, and (**e**) kidney tissues 15 min after injection (hematoxylin–eosin staining, 200 ×).

Pathological results showed that the boundary of the black magnetic gel at the injection site was clear, with no diffusion of the magnetic fluid (Fig. 8). No magnetic fluid was observed in the rest of the lung tissue. The heart, liver, spleen, and kidney tissues retained good histologic morphology and no magnetic fluid was observed.

### Results of long-term localization

#### X-ray observation of the magnetic gel

**Figure 9:**
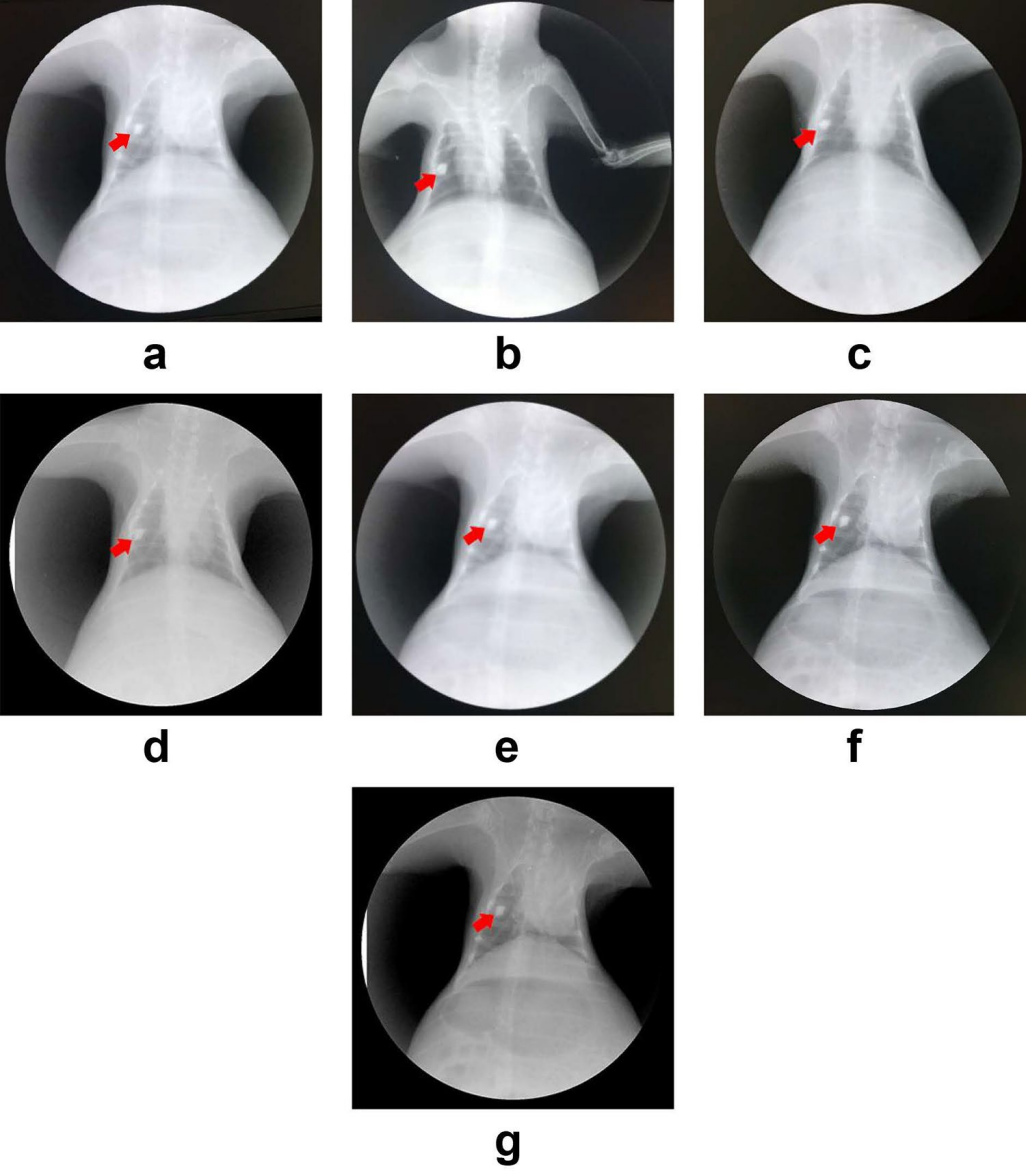
(**a**–**g**) Long-term localization of the magnetic gel in lung tissue, viewed under X-ray, are indicated by a red arrow, days 1–7 after the operation, respectively.

All 12 rabbits were successfully injected with magnetic gel to locate imaginary lung nodules. No complications such as bleeding or pneumothorax occurred during the operation. The condition of the rabbits was good after the operation, with no coughing or dyspnea observed. No diffusion of magnetic fluid or displacement of the magnetic gel was observed under X-ray within 7 days (Fig. 9).

#### Observation of gross specimens

**Figure 10:**
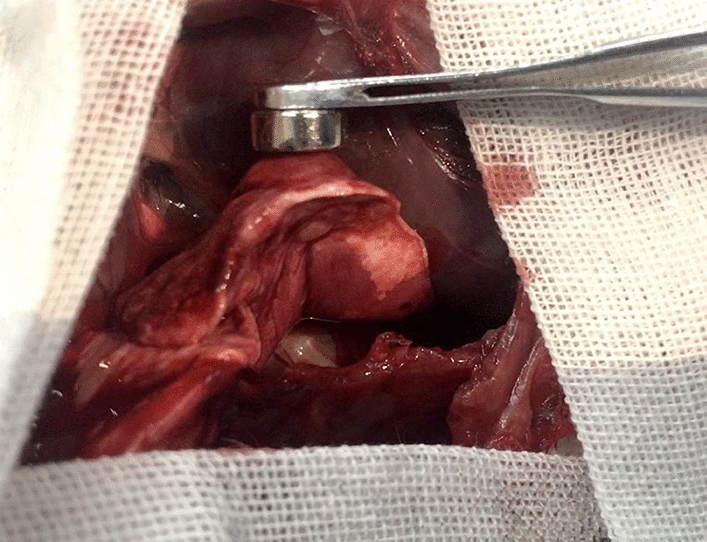
Lung tissue attracted by the pursuit magnet.

On days 1, 3, 5, and 7 after the operation, the rabbits were euthanized and the chest was opened. A pursuit magnet was used to provide an applied magnetic field to the lung surface to observe the localization effect. We observed that the magnetic gel was attracted by the applied magnetic field, and that the local area was involved. The lung tissue where the hypothetical SPN located was protruded from the surface of the lung (Fig. 10).

#### Diffusion of magnetic fluid

**Figure 11:**
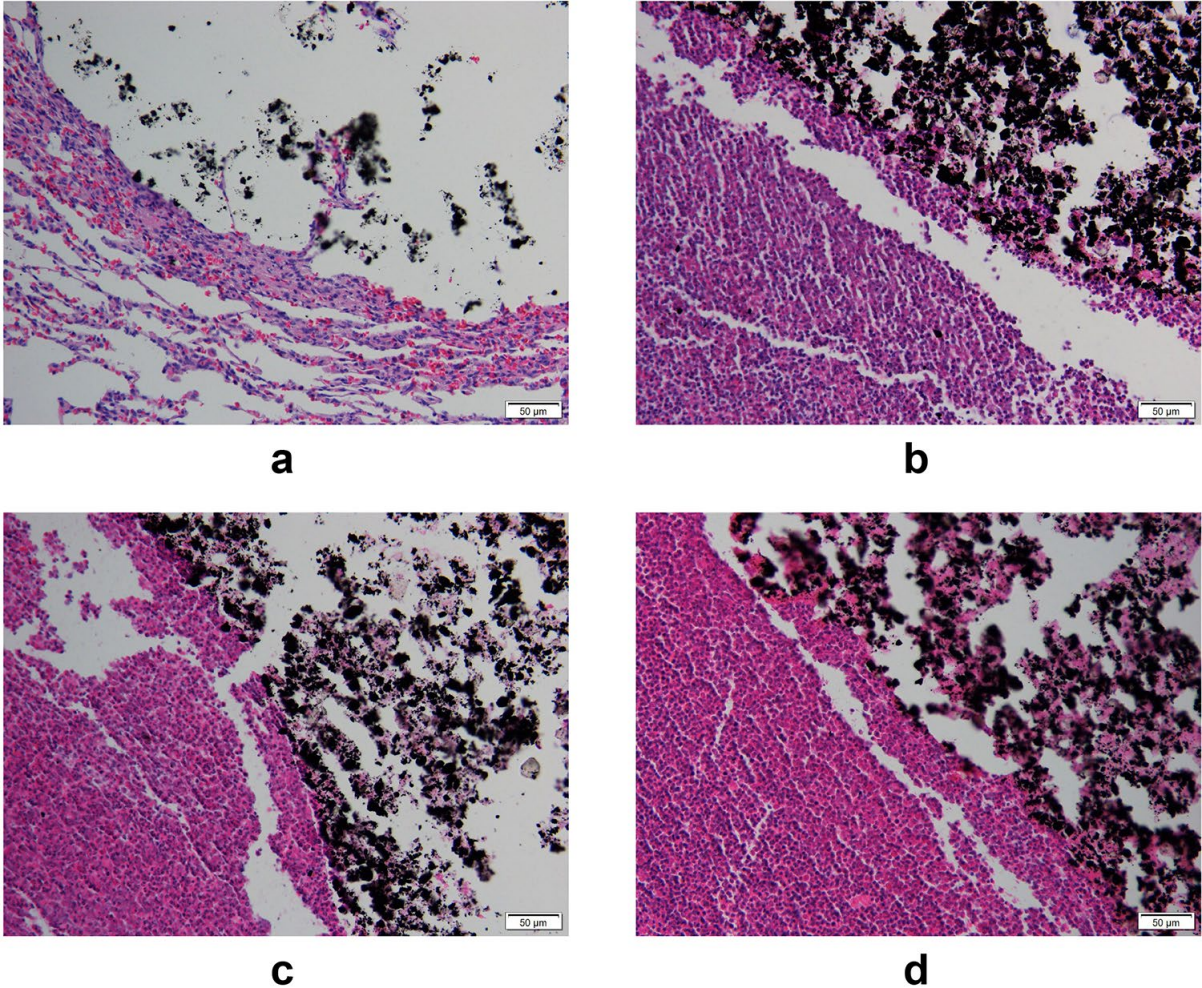
(**a**–**d**) The injection site on postoperative days 1, 3, 5, and 7, respectively (hematoxylin–eosin staining, 200×).

**Figure 12:**
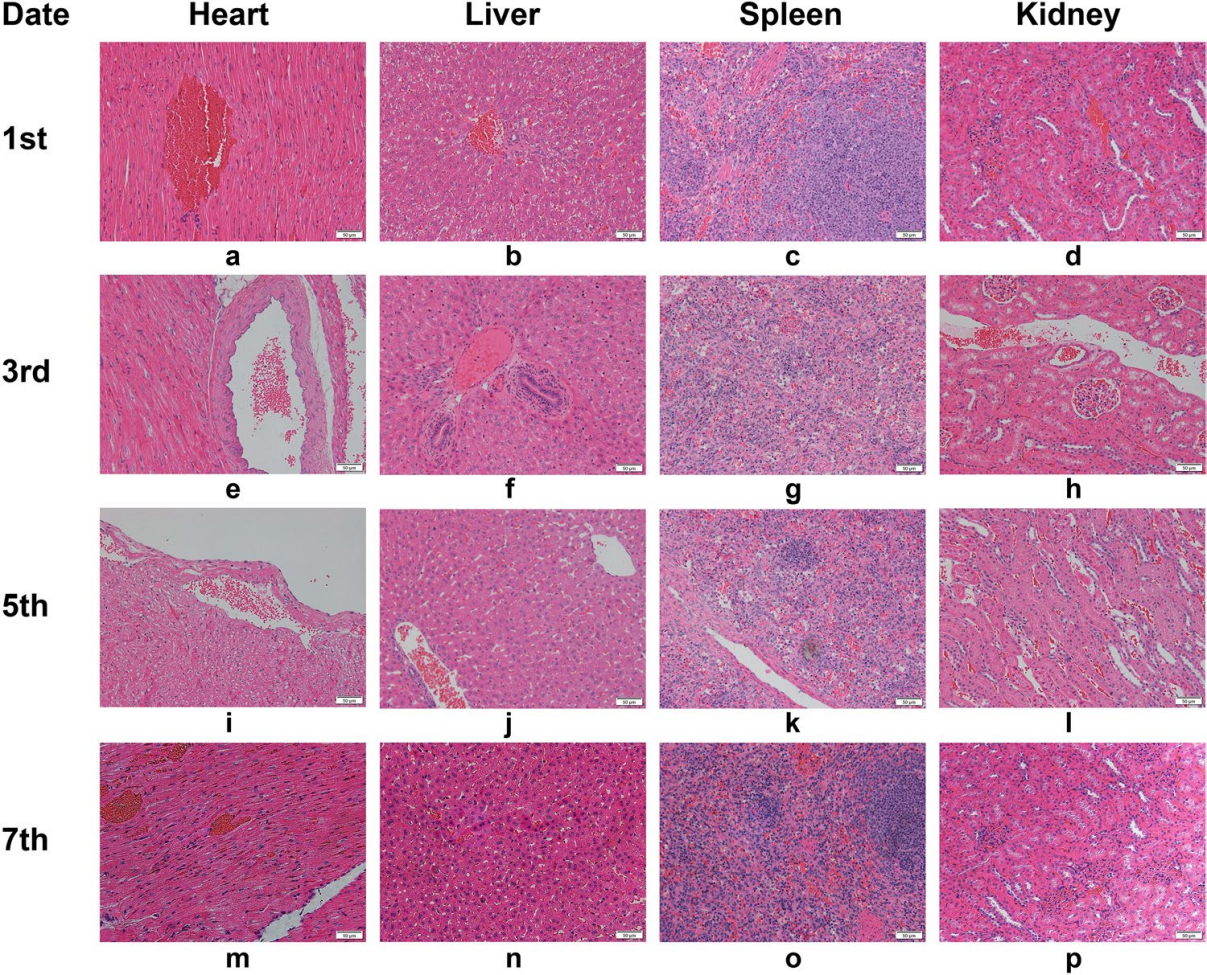
(**a**–**p**) Morphology of tissue at postoperative day 1, 3, 5, and 7. (hematoxylin–eosin staining, 200×).

We excised tissue from the injection site and stained it with hematoxylin–eosin. We observed a clear boundary to the magnetic fluid and no diffusion. At days 1, 3, 5, and 7, no significant difference in the shape of the magnetic fluid was observed; at days 3, 5, and 7, the surrounding lung tissue had varying degrees of fibrosis, and the degree of fibrosis gradually increased over time (Fig. 11). Hematoxylin–eosin stained heart, liver, spleen, and kidney tissues showed good morphology and no magnetic fluid was observed (Fig. 12).

## Conclusion

The method of applying magnetic gel to locate SPNs is safe and feasible. We provide an animal experimental basis for the clinical application of this method. When this method is used for human lung nodule localization, magnetic fluid and calcium gluconate can be injected under the guidance of computed tomography to form gel, the focus can be located with tracking magnet under thoracoscopy, and small lung nodules can be removed with non-magnetic surgical instruments such as titanium alloy.

## Data Availability

All data generated and analysed during this study are included in this published article.

## References

[1] Bai C (2016). Evaluation of pulmonary nodules: Clinical practice consensus guidelines for Asia. Chest.

[2] Su C (2016). From diagnosis to therapy in lung cancer: Management of CT detected pulmonary nodules, a summary of the 2015 Chinese-German Lung Cancer Expert Panel. Transl. Lung Cancer Res..

[3] Horeweg N (2014). Lung cancer probability in patients with CT-detected pulmonary nodules: A prespecified analysis of data from the NELSON trial of low-dose CT screening. Lancet Oncol.

[4] Walter JE (2016). Occurrence and lung cancer probability of new solid nodules at incidence screening with low-dose CT: Analysis of data from the randomised, controlled NELSON trial. Lancet Oncol..

[5] Liu B, Gu C (2020). Expert consensus workshop report: Guidelines for preoperative assisted localization of small pulmonary nodules. J. Cancer Res. Ther..

[6] Lee JW, Park CH, Lee SM, Jeong M, Hur J (2019). Planting seeds into the lung: Image-guided percutaneous localization to guide minimally invasive thoracic surgery. Korean J. Radiol..

[7] Sun S (2020). Application of modified tailed microcoil in preoperative localization of small pulmonary nodules: A retrospective study. Thorac. Cardiovasc. Surg..

[8] Kadeer X, Wang L, Zhang L, Shi W, Chen C (2018). Modified hook-wire placement technique for localizing multiple pulmonary nodules. J. Surg. Oncol..

[9] Su TH, Fan YF, Jin L, He W, Hu LB (2015). CT-guided localization of small pulmonary nodules using adjacent microcoil implantation prior to video-assisted thoracoscopic surgical resection. Eur. Radiol..

[10] Tseng YH (2016). Preoperative computed tomography-guided dye injection to localize multiple lung nodules for video-assisted thoracoscopic surgery. J. Thorac. Dis..

[11] Yan X (2018). Exploration and establishment of magnetic surgery. Chin. Sci. Bull..

[12] Douek M (2014). Sentinel node biopsy using a magnetic tracer versus standard technique: The SentiMAG Multicentre Trial. Ann. Surg. Oncol..

[13] Ling Y (2020). Study of colorectal neoplasms localization based on magnetic tracer technique in an animal model. Chin. J. Dig. Endosc..

[14] Ma J (2020). Application of magnetic tracer technique in labeling and localization of esophageal tumors. China J. Endosc..

[15] Fan Q (2020). Experimental study of stomach tumor localization based on magnetic tracer technique. Chin. J. Gen. Surg..

[16] Siegel RL, Miller KD, Jemal A (2018). Cancer statistics, 2018. CA Cancer J. Clin..

[17] Baldwin DR, Devaraj A (2016). Lung cancer risk in new pulmonary nodules: Implications for CT screening and nodule management. Lancet Oncol..

[18] Lin CW (2019). Computed tomography-guided dual localization with microcoil and patent blue vital dye for deep-seated pulmonary nodules in thoracoscopic surgery. J. Formos. Med. Assoc..

[19] Tsai TM, Hung WT, Lin MW, Hsu HH, Chen JS (2019). Computed tomography-guided dye localization prior to uniportal thoracoscopic surgery for lung nodules: A propensity score matching analysis. J. Formos. Med. Assoc..

[20] Zhong Y (2017). Retrospective evaluation of safety, efficacy and risk factors for pneumothorax in simultaneous localizations of multiple pulmonary nodules using hook wire system. Cardiovasc. Intervent. Radiol..

[21] Li Q, Dai J, Zhang P, Jiang G (2021). Management of pulmonary ground glass nodules: Less is more. Ann. Thorac. Surg..

[22] Liu SQ (2012). Nonsuture anastomosis of arteries and veins using the magnetic pinned-ring device: A histologic and scanning electron microscopic study. Ann. Vasc. Surg..

[23] Colombo M (2012). Biological applications of magnetic nanoparticles. Chem. Soc. Rev..

[24] Das P, Colombo M, Prosperi D (2019). Recent advances in magnetic fluid hyperthermia for cancer therapy. Colloids Surf. B Biointerfaces.

[25] Grizzi I, Braud C, Vert M (1998). Calcium alginate dressings—I. Physico-chemical characterization and effect of sterilization. J Biomater. Sci. Polym. Ed..

[26] Mao D, Li Q, Li D, Tan Y, Che Q (2018). 3D porous poly(ε-caprolactone)/58S bioactive glass–sodium alginate/gelatin hybrid scaffolds prepared by a modified melt molding method for bone tissue engineering. Mater. Des..

[27] Shi X (2016). Microspheres of carboxymethyl chitosan, sodium alginate and collagen for a novel hemostatic in vitro study. J. Biomater. Appl..

[28] Wang H (2019). Characterization, release, and antioxidant activity of curcumin-loaded sodium alginate/ZnO hydrogel beads. Int. J. Biol. Macromol..

[29] Fu C (2017). MoS2 nanosheets encapsulated in sodium alginate microcapsules as microwave embolization agents for large orthotopic transplantation tumor therapy. Nanoscale.

[30] Pankratov Y, Lalo U (2014). Calcium permeability of ligand-gated Ca2+ channels. Eur J Pharmacol.

[31] Karlsson HL, Gustafsson J, Cronholm P, Moller L (2009). Size-dependent toxicity of metal oxide particles—A comparison between nano- and micrometer size. Toxicol Lett.

[32] Braszczok N (2011). In vitro evaluation of magnetic resonance imaging contrast agents for labeling human liver cells: implications for clinical translation. Mol. Imaging Biol..

[33] Vaithilingam V (2016). Noninvasive tracking of encapsulated insulin producing cells labelled with magnetic microspheres by magnetic resonance imaging. J. Diabetes Res..

[34] Vinod E (2019). Comparison of incremental concentrations of micron-sized superparamagnetic iron oxide for labelling articular cartilage derived chondroprogenitors. Acta Histochem..

[35] Dallet L, Stanicki D, Voisin P, Miraux S, Ribot EJ (2021). Micron-sized iron oxide particles for both MRI cell tracking and magnetic fluid hyperthermia treatment. Sci. Rep..

[36] Shapiro EM, Skrtic S, Koretsky AP (2005). Sizing it up: Cellular MRI using micron-sized iron oxide particles. Magn. Reson. Med..

[37] McAteer MA (2008). Magnetic resonance imaging of endothelial adhesion molecules in mouse atherosclerosis using dual-targeted microparticles of iron oxide. Arterioscler. Thromb. Vasc. Biol..

[38] Zhao G (2017). Semi-rigid single hook localization the best method for localizing ground glass opacities during video-assisted thoracoscopic surgery: Re-aerated swine lung experimental and primary clinical results. J. Thorac. Dis..

